# CORESS Feedback: Cases from the Confidential Reporting System for Surgery

**DOI:** 10.1308/rcsann.2025.0069

**Published:** 2025-09-01

**Authors:** HJ Corbett, W Hawkins

**Affiliations:** on behalf of the CORESS Advisory Board

## Abstract

CORESS is an independent charity. We are grateful to those who have provided the material for these reports, some of which comes from NHS England and GIRFT reports. The online reporting form is available via the CORESS website (www.coress.org.uk), which also includes previous Feedback reports. Published cases are acknowledged by a Certificate of Contribution, which may be included in the contributor’s record of continuing professional development or may form part of appraisal or annual review of competence progression portfolio documentation. Contributions from surgeons in training are particularly welcome.

## Total Hip Replacement (THR): Failure to document anatomical variations

### Case 310

Having had a right uncemented total hip replacement using an 8mm femoral stem, a man in his 60s required revision surgery 2 years later because of persistent pain due to loosening and fracture of the femoral stem.

The Claimant alleged that the entry point for introduction of both the rasps and prosthesis had been excessively medial, resulting in an inappropriately small prosthesis being used and implanted in varus. There was subsequent migration and aseptic loosening of the component and failure of the prosthesis. The allegation was not accepted by the operating surgeon, who claimed that he had to use the small prosthesis because of the abnormally excessive curvature of the Claimant's femur.

The case could not be defended at trial, however, as there was found to be insufficient evidence in the operation note to support the surgeon's account. Specifically, there was a lack of detail as to the surgical approach and a lack of explanation in the operative record regarding the rationale for the unusually small prosthesis.

Damages of £15,000 plus costs were paid to settle the claim.

### GIRFT message

Any unusual anatomy and the steps taken to adapt to those anatomical variations should be documented.

### CORESS comments

A comprehensive operation note forms a fundamental component of communication in the continuity of surgical care, and may also provide a means of defence in litigation cases. The Royal College of Surgeons of England has set out in detail the components of a good operation note in the document *Good Surgical Practice.*^1^ Further relevant details are provided in Hoggett’s ‘How to write an operation note’.^2^ The CORESS Advisory Board noted that it was particularly important to document untoward, unusual or adverse occurrences, in the event that documentation is subsequently relied on for medicolegal purposes. Where feasible, a drawing or illustration may significantly enhance a reader’s understanding of the procedure undertaken.

References1.Royal College of Surgeons of England. Good Surgical Practice. https://www.rcseng.ac.uk/standards-and-research/hgsp (cited August 2025).2.Hoggett
L. How to write an operation note. BMJ
2017; **356**: j355.

## Total Hip Replacement (THR): Use of non-compatible implants

### Case 311

A man in his 50s underwent left hip resurfacing using a system that requires a specific match between the head and cup components. To ensure this, the components are packaged with colour coding. During the procedure a 50mm ‘GREY’ head was implanted with a 52mm ‘RED’ cup. This component size mismatch and the associated component ‘colour’ discrepancy was not noted at the time of the surgery. The mismatched components led to early failure of the prosthesis. The resurfacing was revised 2 years later. To compound this error, the additional failure to register the implant with the National Joint Registry meant that this additional check was not introduced.

Damages of £15,000, plus costs, were awarded.

### GIRFT message

Components should be checked by the surgeon and scrub team at the time they are opened and implanted, and a final review and check for compatibility should be made prior to closure. Implant identification data must be submitted to the National Joint Registry. Although helpful, the use of colour coding is not fail-safe particularly when members of the surgical team might be colour blind.

### CORESS comments

It was noted that the Devices branch of the Medicines and Healthcare products Regulatory Agency have investigated colour coding of devices extensively. Problems arise because of different colours being recognised internationally, manufacturers changing colours, and variation in text sizes and labelling on packaging. Universal barcoding may help to reduce incidents, but is not a panacea.

Risks of device or implant mismatch are reduced by triangulation of colours with numerical component sizes, and by dual verification by more than one member of the surgical team. Prior to starting a procedure, checks should be undertaken to ensure matching components and that all relevant component sizes are available.

Implant verification processes are covered in detail in the National Safety Standards for Invasive Procedures (NatSSIPs 2) document.^1^

Reference1.Centre for Perioperative Care. National Safety Standards for Invasive Procedures. https://cpoc.org.uk/guidelines-resources-guidelines/national-safety-standards-invasive-procedures-natssips (cited August 2025).

## Inadequate operative documentation and procedural description, An appendicectomy that wasn’t: case no. 1

### Case 313

Following a week of being unwell, with eventual development of severe lower abdominal pain and rigors, a 32-year-old man underwent laparoscopic intervention. The procedure was listed as laparoscopic appendicectomy. At surgery, a large abscess in the right iliac fossa was drained and washed out. The appendix could not be seen because of the abscess and local inflammation. The operating surgeon failed to document in the operation note that the appendix had not been removed, although the operation was described as a laparoscopic appendicectomy.^1^

Unfortunately, the patient continued to deteriorate because the rest of the clinical team were unaware that the source of the infection remained in situ, and when the patient did not improve, the appendix was not removed in the days immediately following the initial procedure. The patient subsequently required further operations including laparotomy and formation of a temporary stoma.

After a prolonged hospital stay, the patient was left with a large abdominal scar and the need for stoma reversal. A medicolegal claim was successful. The washout and drainage of the abscess was advised as reasonable by the defence expert, but failure to record that the appendix was left in situ was a breach of duty.

### Reporter’s comments

Document if there are any intraoperative complications or unexpected findings, and if part of the original planned procedure is not performed and the rationale for this. Ensure that any intraoperative decision making is communicated to the patient and clinical team with an explanation behind the change in procedure performed and document that this was communicated to the patient and that any questions have been answered.

### CORESS comments

CORESS noted that irrespective of the operation, continued deterioration of the patient should have been recognised and acted upon. This may have required updated imaging. The sign-out check would have provided an opportunity to confirm the correct nature of the procedure with the team and to ensure that this was correct in the operative record.

Reference1.
GIRFT, RCS and ASGBI. *Best Practice for Laparoscopic Appendicectomy Documentation*. https://gettingitrightfirsttime.co.uk/wp-content/uploads/2022/09/GIRFT-best-practice-lap-appendicectomy-Final-20220830.pdf (cited August 2025).


## An appendicectomy that wasn’t: case no. 2

### Case 320

A 25-year-old female presented with right iliac fossa (RIF) pain and a high temperature. Appendicitis was diagnosed, a laparoscopic procedure performed and the patient was prescribed postoperative antibiotics. The patient was discharged on day 3 postoperatively, but readmitted on day 6 with pain, fever and high C reactive protein (CRP) of 320. A computed tomography (CT) scan was performed and an abscess identified in the RIF which was drained percutaneously. The patient had a postoperative ileus and total parenteral nutrition (TPN) was started. She recovered slowly, requiring TPN for two weeks. A month after discharge the patient attended the outpatient clinic complaining ongoing pain. A further CT scan was performed which surprisingly identified an inflamed appendix. The histology of the tissue removed at the initial operation was reviewed and the tissue was found to be inflamed fat, with no appendicular tissue. Review of the previous CT scan also identified the appendix.

**Figure 1:**
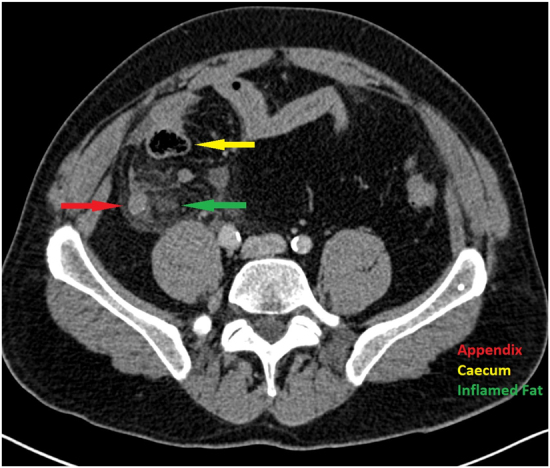
CT scan showing appendix and inflamed fat. Finding the appendix during surgery can be a challenge when inflammation obscures anatomical planes.

### Reporter’s comments

The experience of the surgeon is important when treating appendicitis and surgeons in training need to be supported in all cases. CT scan reporting was misled by the clinical information and the histology department could have alerted the surgical team that the specimen did not confirm removal of the appendix. As usual the hardest thing about appendicitis is finding it!

### CORESS comments

Appendicectomy can be a challenging operation, especially when significant inflammation obscures anatomical planes (Figure 1). Although the difficulty of the initial operation may explain the failure to remove the appendix, there were two opportunities to identify the problem, both of which were missed. First, the histopathology report was clear that the appendix was not within the analysed specimen, but the report was not seen in a timely manner. Although the system by which abnormal results were flagged to the clinical team was presumably inadequate, this is a reminder that relevant results should be checked when a patient is not making the expected recovery. Second, the report of the initial CT was, however, incorrect. This represents a scenario of ‘seeing what you expect to see’; that is, assuming the appendix was absent based on the clinical history. The maxim ‘assume nothing’ seems pertinent in this case.

## Consent issues and failure to treat low parathyroid hormone post-thyroidectomy

### Case 314

A patient with a family history of thyroid cancer was referred to hospital for investigation of a lump in her neck. Ultrasound and fine-needle aspiration cytology suggested a benign cyst. A computed tomography scan showed some early narrowing of the trachea on the left and the left thyroid lobe appeared larger. The possibility of surgery (left hemi-thyroidectomy or total thyroidectomy) was discussed, and the patient elected to proceed with a total thyroidectomy.^1^

The consent form stated that the intended benefits were to relieve compressive symptoms, and the risks were noted as scarring, bleeding, infection, hypocalcaemia, necessity for hormone supplements, hoarse voice, loss of voice, airway compromise and tracheostomy. A total thyroidectomy was performed. Postoperatively, while in hospital, the patient developed respiratory problems and carpopedal spasm, which was not recognised promptly.

Allegations were raised that there was a failure to obtain informed consent, that a partial thyroidectomy should have been performed and that there was a failure to monitor for hypoparathyroidism postoperatively. The patient claimed that had she been consented properly, she would have opted for a partial thyroidectomy and if her condition had been monitored appropriately, she would have avoided severe hypocalcaemia and tetanic muscular spasms. Eventually, the trust settled a claim for damages.

### Reporter’s comments

Permanent hypoparathyroidism is a recognised risk in thyroidectomy, although there was no definite evidence that the patient had permanent hypoparathyroidism. However, areas of vulnerability identified for the trust were in relation to bruising of the parathyroid glands during the thyroidectomy, and that the low parathyroid hormone was not treated promptly postoperatively with calcium supplementation and repeat monitoring prior to discharge. The case highlights the importance of documenting parathyroid hormone and calcium levels after thyroidectomy, before discharge from hospital.

### CORESS comments

Although a formal consent form is helpful in documenting risks discussed with the patient, a record of the consent discussions undertaken in the outpatient department and patient involvement in the clinical decisions, copied to both patient and general practitioner, might have been useful in this case.

Reference1.
GIFRT, RCS and BAETS. Best Practice for Thyroidectomy Documentation. https://gettingitrightfirsttime.co.uk/wp-content/uploads/2022/09/GIRFT-best-practice -thyroidectomy-Final-20220830.pdf.


## Medication mismanagement: continuation of sodium–glucose co-transporter-2 inhibitors when Nil by mouth (NBM) causing euglycaemic diabetic ketoacidosis

### Case 319

A 57-year-old woman with a background history of type 2 diabetes mellitus (T2DM) and a Roux-en-Y gastric bypass was admitted with abdominal pain and jaundice. An MRCP was performed, which showed cholecystitis, and a dilated common bile duct (CBD) with distal stones. The patient underwent laparoscopic cholecystectomy with exploration of the CBD, which was repaired leaving a T-tube in the duct. Two days postoperatively, she became drowsy and delirious, with a Glasgow Coma Score of 9. She developed an acute kidney injury, metabolic acidosis and ketosis. At this point it was noted that the patient had continued to receive empagliflozin since her admission and this had led to euglycaemic ketoacidosis.

### Reporter’s comments

Sodium–glucose co-transporter 2 (SGLT2) inhibitors (such as canagliflozin, dapagliflozin, empagliflozin and ertugliflozin) are a relatively new class of oral drugs for the management of T2DM. We should be aware of potential side effects of these medications. Surgical teams should work closely, where possible, with pharmacy teams to ensure medicine reconciliation, prior to admission, to reduce medication errors. It would be helpful to introduce systems for people with diabetes to report changes to their medication between their preoperative assessment and date of surgery. As per Centre for Perioperative Care and Academy of Medical Royal Colleges guidelines for perioperative care for people with diabetes mellitus, the following are recommended: first, SGLT2 inhibitors should be withheld 48–72h prior to all major surgeries, and plasma glucose levels should be closely monitored perioperatively; second, there should be vigilant postoperative assessment for diabetic ketoacidosis, even with normal plasma glucose; and third, these drugs should only be restarted when the patient is eating and drinking normally postoperatively.

### CORESS comments

Awareness of serious side effects of medication is vital, especially in emergency care when preoperative planning is not possible. The British Obesity and Metabolic Surgery Society (BOMSS), issued a Patient Safety Alert regarding the risk of harm from the use of SGLT-2 inhibitors in patients undergoing bariatric surgery in March 2022, which outlines the importance of understanding the risks associated with this class of medication. Patients may not remember or understand the risk, especially if they are on a number of different medications so increased awareness among healthcare professionals is important. Safety alerts embedded into digital prescribing systems should alert prescribers to the risk of euglycaemic diabetic ketoacidosis in fasting patients.

## Fatality after delayed recognition of wrong end stoma formation: ‘failure to rescue’

### Case 321

A 60-year-old woman with advanced metastatic endometrial cancer was scheduled to have (and consented for) a laparoscopic defunctioning loop colostomy as a palliative procedure, to relieve symptoms from a rectovaginal fistula. The procedure was performed by a senior staff grade surgeon under the supervision of a consultant colorectal surgeon who was in theatre but not scrubbed. A decision was made by the consultant to perform an end-colostomy rather than a loop colostomy when the procedure was underway. This was questioned by the operating surgeon but was nevertheless undertaken. No post-procedure checks were carried out to confirm that the correct end of the stoma had been exteriorised. Unfortunately, the proximal end of the descending colon had been stapled and the distal end of the colon, proximal to the fistula, brought out as the stoma. The patient became distended almost immediately after surgery, but recognition of what was effectively wrong-site surgery was delayed for 7 days because there had been some collection of mucous type fluid in the stoma bag. When a computed tomography (CT) scan was eventually requested and the error detected, the patient returned to theatre as an emergency and the proximal colon exteriorised. The patient returned to the intensive care unit but rapidly deteriorated and died.

### Reporter’s and CORESS comments

The decision to operate was made in conjunction with the patient and her family, and the patient had been discussed at a multidisciplinary team meeting. This was deemed to be good practice. A firm decision should have been made on the type of colostomy (loop vs end) prior to the procedure, avoiding confusion and debate once the operation had started. The patient was noted to be very high risk. The procedure should have been performed by the consultant, or at least with the consultant directly assisting the operating surgeon. Post-procedural confirmation that the correct end of bowel had been exteriorised should have been instigated in line with national recommendations. The failure of the stoma to function properly, abdominal distension and raised inflammatory markers should have triggered an early request for a CT scan of the abdomen. Postoperative patient review should have been undertaken by a consultant or equivalent senior surgeon.

## Wrong side consent form

### Case 322

A 15-year-old boy with a solitary left testis was referred for consideration of a testicular prosthesis. A testicular remnant had been removed from the right groin in infancy, confirming a diagnosis of testicular regression. He was seen in clinic with his parents on two occasions; at the first visit he was examined and found to have a normal post-pubertal testis in the left hemiscrotum. He received counselling regarding the potential complications associated with insertion of a testicular prosthesis and a further review was advised to allow the patient time to think about whether he wanted to go ahead. At the second review the patient advised that he wanted to have a prosthesis inserted. As he was considered to be ‘Gillick competent’, a consent form 1 was completed using an electronic consent platform and the patient was keen to sign the form ‘there and then’ even though the option of accessing the form remotely was offered. On the day of surgery, he was seen by an astute trainee who examined the patient, reviewed the notes and the operating list, and noted that the consent form stated insertion of a left-sided prosthesis instead of the right. The incorrect form was cancelled, a new form created and the procedure completed on the correct side.

### Reporter’s comments

The consultant made an error when ticking the box for the side on the electronic consent form and this was not noticed by the patient, who may have been embarrassed and keen to complete the consultation promptly, although it may be that having always had a solitary testis, he was not sure which side it was on. The registrar was not planning to tell the consultant of their error, although the consultant spotted it because there were two consent forms visible in the electronic system at the ‘sign in’. This was a valuable lesson for both the consultant and the registrar because ‘covering up’ a near-miss is a missed opportunity to learn. Of note, the electronic consent platform has since improved the ‘visibility’ of which side is selected owing to concerns raised by other users at our trust.

### CORESS comments

The revised National Safety Standards for Invasive Procedures (NatSSIPS2) involve eight sequential steps and highlight the importance of communication and situational awareness, both of which are relevant to this case.^1^ The ‘Consent, Procedure Verification and Site Marking’ step must be undertaken by a person knowledgeable with the procedure, with access to the medical records and should involve the patient. The trainee made a ‘good catch’ because the patient themselves may have been unsure of the side of the procedure, distracted by the approaching procedure or reluctant to speak up. Although the side error may have been obvious before an incision was made in this scenario, identification of the error at the earliest possible stage benefits the entire team. The value of reporting near-miss incidents such as this should not be underestimated.

Reference1.Centre for Perioperative Care. National Safety Standards for Invasive Procedures. https://cpoc.org.uk/guidelines-resources-guidelines/national-safety-standards-invasive-procedures-natssips (cited August 2025).
